# Supplementary material for: Using Large Language Models to Measure Symptom Severity Scores in Patients At-Risk for Schizophrenia
Source: Schizophr Bull. 2026 Aug 24;52(5):sbag084. doi: 10.1093/schbul/sbag084 (PMC13502897; doi:10.1093/schbul/sbag084)
Supplement: Supplementary file 1 [file supplementary_material_sbag084.zip › amp_supplement.pdf]

Supplementary Materials

Using Large Language Models to Measure Symptom Severity Scores in Patients At-Risk for Schizophrenia

Index

A. Full prompt supplied to LLM
B. Subscores predicted by LLM - attachment S1.csv
C. Explanations provided for subscore ratings - attachment S2.csv

A. Full prompt supplied to LLM

For the following clinical interview transcript, provide an estimated score for each of the 24 items on the expanded Brief Psychiatric Rating Scale, using the following reference guide.

BRIEF PSYCHIATRIC RATING SCALE (BPRS) Expanded Version

SCALE ITEMS AND ANCHOR POINTS

Rate items 1 - 14 on the basis of patient's self report. Note items 7, 12, and 13 are also rated on the basis of observed behavior. Items 15 - 24 are rated on the basis of observed behavior and speech.

1. SOMATIC CONCERN: Degree of concern over present bodily health. Rate the degree to which physical health is perceived as a problem by the patient, whether complaints have realistic bases or not. Somatic delusions should be rated in the severe range with or without somatic concern. Note: Be sure to assess the degree of impairment due to somatic concerns only and not other symptoms, e.g., depression. In addition, if the subject rates a "6" or "7" due to somatic delusions, then you must rate Unusual Thought Content at least a "4" or above.

Have you been concerned about your physical health? Have you had any physical illness or seen a medical doctor lately? (What does your doctor say is wrong? How serious is it?
Has anything changed regarding your appearance?
Has it interfered with your ability to perform your usual activities and/or work?
Did you ever feel that parts of your body had changed or stopped working?

[If patient reports any somatic concerns/delusions, ask the following]:

How often are you concerned about [use patient's description]?

Have you expressed any of these concerns to others?

1 Not Present

2 Very Mild
Occasional somatic concerns that tend to be kept to self.

3 Mild
Occasional somatic concerns that tend to be voiced to others (e.g., family, physician).

4 Moderate
Frequent expressions of somatic concern or exaggerations of existing ills OR some preoccupation, but no impairment in functioning. Not delusional.

5 Moderately Severe
Frequent expressions of somatic concern or exaggeration of existing ills OR some preoccupation and moderate impairment of functioning. Not delusional

6 Severe
Preoccupation with somatic complaints with much impairment in functioning OR somatic delusions without acting on them or disclosing to others.

7 Extremely Severe
Preoccupation with somatic complaints with severe impairment in functioning OR somatic delusions that tend to be acted on or disclosed to others.

2. ANXIETY: Reported apprehension, tension, fear, panic or worry. Rate only the patient's statements, not observed anxiety which is rated under TENSION.

Have you been worried a lot during [mention time frame]? Have you been nerv-ous or apprehensive? (What do you worry about?) Are you concerned about anything? How about finances or the future? When you are feeling nervous, do your palms sweat or does your heart beat fast (or shortness of breath, trembling, choking)?

[If patient reports anxiety or autonomic accompaniment, ask the following]:

How much of the time have you been [use patient's description]?
Has it interfered with your ability to perform your usual activities/work?

1 Not Present

2 Very Mild
Reports some discomfort due to worry OR infrequent worries that occur more than usual for most normal individuals.

3 Mild
Worried frequently but can readily turn attention to other things.

4 Moderate
Worried most of the time and cannot turn attention to other things easily but no impairment in functioning OR occasional anxiety with autonomic accompaniment but no impairment in functioning.

5 Moderately Severe
Frequent, but not daily, periods of anxiety with autonomic accompaniment OR some areas of functioning are disrupted by anxiety or worry.

6 Severe
Anxiety with autonomic accompaniment daily but not persisting throughout the day OR many areas of functioning are disrupted by anxiety or constant worry.

7 Extremely Severe
Anxiety with autonomic accompaniment persisting throughout the day OR most areas of functioning are disrupted by anxiety or constant worry.

3. DEPRESSION: Include sadness, unhappiness, anhedonia, and preoccupation with depressing topics (can't attend to TV or conversations due to depression), hopelessness, loss of self esteem (dissatisfied or disgusted with self or feelings of

worthlessness). Do not include vegetative symptoms, e.g., motor retardation, early waking, or the amotivation that accompanies the deficit syndrome.

How has your mood been recently? Have you felt depressed (sad, down, unhappy as if you didn't care)? Are you able to switch your attention to more pleasant topics when you want to? Do you find that you have lost interest in or get less pleasure from things you used to enjoy, like family, friends, hobbies, watching TV, eating?

[If subject reports feelings of depression, ask the following]:

How long do these feelings last? Has it interfered with your ability to perform your usual activities/work?

1 Not Present

2 Very Mild
Occasionally feels sad, unhappy or depressed.

3 Mild
Frequently feels sad or unhappy but can readily turn attention to other things.

4 Moderate
Frequent periods of feeling very sad, unhappy, moderately depressed, but able to function with extra effort.

5 Moderately Severe
Frequent, but not daily, periods of deep depression OR some areas of functioning are disrupted by depression.

6 Severe
Deeply depressed daily but not persisting throughout the day OR many areas of functioning are disrupted by depression.

7 Extremely Severe

Deeply depressed daily OR most areas of functioning are disrupted by depression.

4. SUICIDALITY: Expressed desire, intent or actions to harm or kill self.

Have you felt that life wasn't worth living? Have you thought about harming or killing yourself? Have you felt tired of living or as though you would be better off dead? Have you ever felt like ending it all?

[If patient reports suicidal ideation, ask the following]:

How often have you thought about [use patient's description]?
Did you (Do you) have a specific plan?

1 Not Present

2 Very Mild
Occasional feelings of being tired of living. No overt suicidal thoughts.

3 Mild
Occasional suicidal thoughts without intent or specific plan OR he/she feels they would be better off dead.

4 Moderate
Suicidal thoughts frequent without intent or plan.

5 Moderately Severe
Many fantasies of suicide by various methods. May seriously consider making an attempt with specific time and plan OR impulsive suicide attempt using non lethal method or in full view of potential saviors.

6 Severe
Clearly wants to kill self. Searches for appropriate means and time, OR potentially serious suicide attempt with patient knowledge of possible rescue.

7 Extremely Severe

Specific suicidal plan and intent (e.g., "as soon as ________ I will do it by doing X"), OR suicide attempt characterized by plan patient thought was lethal or attempt in secluded environment.

5. GUILT: Over concern or remorse for past behavior. Rate only patient's statements, do not infer guilt feelings from depression, anxiety, or neurotic defenses. Note: If the subject rates a "6" or "7" due to delusions of guilt, then you must rate Unusual Thought Content at least a "4" or above depending on level of preoccupation and impairment.

Is there anything you feel guilty about? Have you been thinking about past problems? Do you tend to blame yourself for things that have happened?
Have you done anything you're still ashamed of?

[If patient reports guilt/remorse/delusions, ask the following]:

How often have you been thinking about [use patient's description]?
Have you disclosed your feelings of guilt to others?

1 Not Present

2 Very Mild
Concerned about having failed someone or at something but not preoccupied. Can shift thoughts to other matters easily.

3 Mild
Concerned about having failed someone or at something with some preoccupation. Tends to voice guilt to others.

4 Moderate
Disproportionate preoccupation with guilt, having done wrong, injured others by doing or failing to do something, but can readily turn attention to other things.

5 Moderately Severe

Preoccupation with guilt, having failed someone or at something, can turn attention to other things, but only with great effort. Not delusional.

6 Severe
Delusional guilt OR unreasonable self reproach very out of proportion to circumstances. Moderate preoccupation present.

7 Extremely Severe
Delusional guilt OR unreasonable self reproach grossly out of proportion to circumstances. Subject is very preoccupied with guilt and is likely to disclose to others or act on delusions.

6. HOSTILITY: Animosity, contempt, belligerence, threats, arguments, tantrums, property destruction, fights and any other expression of hostile attitudes or actions. Do not infer hostility from neurotic defenses, anxiety or somatic complaints. Do not include incidents of appropriate anger or obvious self defense.

How have you been getting along with people (family, co workers, etc.)?
Have you been irritable or grumpy lately? (How do you show it? Do you keep it to yourself?) Were you ever so irritable that you would shout at people or start fights or arguments? (Have you found yourself yelling at people you didn't know?) Have you hit anyone recently?

1 Not Present

2 Very Mild
Irritable or grumpy, but not overtly expressed.

3 Mild
Argumentative or sarcastic.

4 Moderate
Overtly angry on several occasions OR yelled at others excessively.

5 Moderately Severe

Has threatened, slammed about or thrown things.

6 Severe
Has assaulted others but with no harm likely, e.g., slapped or pushed, OR destroyed property, e.g., knocked over furniture, broken windows.

7 Extremely Severe
Has attacked others with definite possibility of harming them or with actual harm, e.g., assault with hammer or weapon.

7. ELEVATED MOOD: A pervasive, sustained and exaggerated feeling of well being, cheerfulness, euphoria (implying a pathological mood), optimism that is out of proportion to the circumstances. Do not infer elation from increased activity or from grandiose statements alone.

Have you felt so good or high that other people thought that you were not your normal self? Have you been feeling cheerful and "on top of the world" without any reason?

[If patient reports elevated mood/euphoria, ask the following]:

Did it seem like more than just feeling good? How long did that last?

1 Not Present

2 Very Mild
Seems to be very happy, cheerful without much reason.

3 Mild
Some unaccountable feelings of well being that persist.

4 Moderate
Reports excessive or unrealistic feelings of well being, cheerful-ness, confidence or optimism inappropriate to circumstances, some of the time. May frequently joke,

smile, be giddy or overly enthusiastic OR few instances of marked elevated mood with euphoria.

5 Moderately Severe
Reports excessive or unrealistic feelings of well being, confidence or optimism inappropriate to circumstances much of the time. May describe feeling on top of the world," "like everything is falling into place," or "better than ever before," OR several instances of marked elevated mood with euphoria.

6 Severe
Reports many instances of marked elevated mood with euphoria OR mood definitely elevated almost constantly throughout interview and inappropriate to content

7 Extremely Severe
Patient reports being elated or appears almost intoxicated, laugh-ing, joking, giggling, constantly euphoric, feeling invulnerable, all inappropriate to immediate circumstances.

8. GRANDIOSITY: Exaggerated self opinion, self enhancing conviction of special abilities or powers or identity as someone rich or famous. Rate only patient's statements about himself, not his demeanor. Note: If the subject rates a "6" or "7" due to grandiose delusions, you must rate Unusual Thought Content at least a "4" or above.

Is there anything special about you? Do you have any special abilities or powers? Have you thought that you might be somebody rich or famous?

[If the patient reports any grandiose ideas/delusions, ask the following]:

How often have you been thinking about [use patient's description]? Have you told anyone about what you have been thinking? Have you acted on any of these ideas?

1 Not Present

2 Very Mild
Feels great and denies obvious problems, but not unrealistic.

3 Mild
Exaggerated self opinion beyond abilities and training.

4 Moderate
Inappropriate boastfulness claims to be brilliant, insightful, or gifted beyond realistic proportions, but rarely self discloses or acts on these inflated self concepts. Does not claim that grandiose accomplishments have actually occurred.

5 Moderately Severe
Same as 4 but often self discloses and acts on these grandiose ideas. May have doubts about the reality of the grandiose ideas. Not delusional.

6 Severe
Delusional claims to have special powers like ESP, to have millions of dollars, invented new machines, worked at jobs when it is known that he was never employed in these capacities, be Jesus Christ, or the President. Patient may not be very preoccupied.

7 Extremely Severe
Delusional - Same as 6 but subject seems very preoccupied and tends to disclose or act on grandiose delusions.

9. SUSPICIOUSNESS: Expressed or apparent belief that other persons have acted maliciously or with discriminatory intent. Include persecution by supernatural or other nonhuman agencies (e.g., the devil). Note: Ratings of "3" or above should also be rated under Unusual Thought Content.

Do you ever feel uncomfortable in public? Does it seem as though others are watching you? Are you concerned about anyone's intentions toward you? Is anyone going out of their way to give you a hard time, or trying to hurt you? Do you feel in any danger?

[If patient reports any persecutory ideas/delusions, ask the following]:

How often have you been concerned that [use patient's description]? Have you told anyone about these experiences?

1 Not Present

2 Very Mild
Seems on guard. Reluctant to respond to some "personal" questions. Reports being overly self conscious in public.

3 Mild
Describes incidents in which others have harmed or wanted to harm him/her that sound plausible. Patient feels as if others are watching, laughing, or criticizing him/her in public, but this occurs only occasionally or rarely. Little or no preoccupation.

4 Moderate
Says others are talking about him/her maliciously, have negative intentions, or may harm him/her. Beyond the likelihood of plausibility, but not delusional. Incidents of suspected persecution occur occasionally (less than once per week) with some preoccupation.

5 Moderately Severe
Same as 4, but incidents occur frequently, such as more than once per week. Patient is moderately preoccupied with ideas of persecution OR patient reports persecutory delusions expressed with much doubt (e.g., partial delusion).

6 Severe
Delusional - speaks of Mafia plots, the FBI, or others poisoning his/her food, persecution by supernatural forces.

7 Extremely Severe
Same as 6, but the beliefs are bizarre or more preoccupying. Patient tends to disclose or act on persecutory delusions.

10. HALLUCINATIONS: Reports of perceptual experiences in the absence of relevant external stimuli. When rating degree to which functioning is disrupted by hal-lucinations, include preoccupation with the content and experience of the hal-lucinations, as well as functioning disrupted by acting out on the hallucinatory content (e.g., engaging in deviant behavior due to command hallucinations). Include thoughts aloud ("gedankenlautwerden") or pseudohallucinations (e.g., hears a voice inside head) if a voice quality is present.

Do you ever seem to hear your name being called? Have you heard any sounds or people talking to you or about you when there has been nobody around? [If hears voices]: What does the voice/voices say? Did it have a voice quality? Do you ever have visions or see things that others do not see? What about smell odors that others do not smell?

[If the patient reports hallucinations, ask the following]:

Have these experiences interfered with your ability to perform your usual activities/work? How do you explain them? How often do they occur?

1 Not Present

2 Very Mild
While resting or going to sleep, sees visions, smells odors, or hears voices, sounds or whispers in the absence of external stimulation, but no impairment in functioning.

3 Mild
While in a clear state of consciousness, hears a voice calling the subjects name, experiences non verbal auditory hallucinations (e.g., sounds or whispers), formless visual hallucinations, or has sensory experiences in the presence of a modality relevant stimulus (e.g., visual illusions) infrequently (e.g., 1 2 times per week) and with no functional impairment.

4 Moderate

Occasional verbal, visual, gustatory, olfactory, or tactile hallucinations with no functional impairment OR non verbal auditory hallucinations/visual illusions more than infrequently or with impairment.

5 Moderately Severe
Experiences daily hallucinations OR some areas of functioning are disrupted by hallucinations.

6 Severe
Experiences verbal or visual hallucinations several times a day OR many areas of functioning are disrupted by these hallucinations.

7 Extremely Severe
Persistent verbal or visual hallucinations throughout the day OR most areas of functioning are disrupted by these hallucinations.

11. UNUSUAL THOUGHT CONTENT: Unusual, odd, strange or bizarre thought content. Rate the degree of unusualness, not the degree of disorganization of speech. Delusions are patently absurd, clearly false or bizarre ideas that are expressed with full conviction. Consider the patient to have full conviction if he/she has acted as though the delusional belief were true. Ideas of reference/persecution can be differentiated from delusions in that ideas are expressed with much doubt and contain more elements of reality. Include thought insertion, withdrawal and broadcast. Include grandiose, somatic and persecutory delusions even if rated elsewhere. Note: if Somatic Concern, Guilt, Suspiciousness, or Grandiosity are rated "6" or "7" due to delusions, then Unusual Thought Content must be rated a "4" or above.

Have you been receiving any special messages from people or from the way things are arranged around you? Have you seen any references to yourself on TV or in the newspapers? Can anyone read your mind? Do you have a special relationship with God? Is anything like electricity, X rays, or radio waves affecting you? Are thoughts put into your head that are not your own? Have you felt that you were under the control of another person or force?

[If patient reports any odd ideas/delusions, ask the following]:

How often do you think about [use patient's description]? Have you told anyone about these experiences? How do you explain the things that have been happening [specify]?

1 Not Present

2 Very Mild
Ideas of reference (people may stare or may laugh at him), ideas of persecution (people may mistreat him). Unusual beliefs in psychic powers, spirits, UFO's, or unrealistic beliefs in one's own abilities. Not strongly held. Some doubt.

3 Mild
Same as 2, but degree of reality distortion is more severe as indicated by highly unusual ideas or greater conviction. Content may be typical of delusions (even bizarre), but without full conviction. The delusion does not seem to have fully formed, but is considered as one possible explanation for an unusual experience.

4 Moderate
Delusion present but no preoccupation or functional impairment. May be an encapsulated delusion or a firmly endorsed absurd belief about past delusional circumstances.

5 Moderately Severe
Full delusion(s) present with some preoccupation OR some areas of functioning disrupted by delusional thinking.

6 Severe
Full delusion(s) present with much preoccupation OR many areas of functioning are disrupted by delusional thinking.

7 Extremely Severe
Full delusions present with almost total preoccupation OR most areas of functioning are disrupted by delusional thinking.

Rate items 12 - 13 on the basis of patient's self report and observed behavior.

12. BIZARRE BEHAVIOR: Reports of behaviors which are odd, unusual, or psychotically criminal. Not limited to interview period. Include inappropriate sexual behavior and inappropriate affect.

Have you done anything that has attracted the attention of others?
Have you done anything that could have gotten you into trouble with the police?
Have you done anything that seemed unusual or disturbing to others?

1 Not Present

2 Very Mild
Slightly odd or eccentric public behavior, e.g., occasionally giggles to self, fails to make appropriate eye contact, that does not seem to attract the attention of others OR unusual behavior conducted in private, e.g., innocuous rituals, that would not attract the attention of others.

3 Mild
Noticeably peculiar public behavior, e.g., inappropriately loud talking, makes inappropriate eye contact, OR private behavior that occasionally, but not always, attracts the attention of others, e.g., hoards food, conducts unusual rituals, wears gloves indoors.

4 Moderate
Clearly bizarre behavior that attracts or would attract (if done privately) the attention or concern of others, but with no corrective intervention necessary. Behavior occurs occasionally, e.g., fixated staring into space for several minutes, talks back to voices once, in appropriate giggling/laughter on 1 2 occasions, talking loudly to self.

5 Moderately Severe
Clearly bizarre behavior that attracts or would attract (if done privately) the attention of others or the authorities, e.g., fixated staring in a socially disruptive

way, frequent inappropriate giggling/laughter, occasionally responds to voices, or eats non-foods.

6 Severe
Bizarre behavior that attracts attention of others and intervention by authorities, e.g., directing traffic, public nudity, staring into space for long periods, carrying on a conversation with hallucinations, frequent inappropriate giggling/laughter.

7 Extremely Severe
Serious crimes committed in a bizarre way that attracts the attention of others and the control of authorities e.g., sets fires and stares at flames OR almost constant bizarre behavior, e.g., inappropriate giggling/laughter, responds only to hallucinations and cannot be engaged in interaction.

13. SELF NEGLECT: Hygiene, appearance, or eating behavior below usual expectations, below socially acceptable standards, or life threatening.

How has your grooming been lately? How often do you change your clothes? How often do you take showers? Has anyone (parents/staff) complained about your grooming or dress? Do you eat regular meals?

1 Not Present

2 Very Mild
Hygiene/appearance slightly below usual community standards, e.g., shirt out of pants, buttons unbuttoned, shoelaces untied, but no social or medical consequences.

3 Mild
Hygiene/appearance occasionally below usual community standards, e.g., irregular bathing, clothing is stained, hair uncombed, occasionally skips an important meal. No social or medical consequences.

4 Moderate

Hygiene/appearance is noticeably below usual community standards, e.g., fails to bathe or change clothes, clothing very soiled, hair unkempt, needs prompting, noticeable by others OR irregular eating and drinking with minimal medical concerns and consequences.

5 Moderately Severe
Several areas of hygiene/appearance are below usual community standards OR poor grooming draws criticism by others, and requires regular prompting. Eating or hydration is irregular and poor, causing some medical problems.

6 Severe
Many areas of hygiene/appearance are below usual community standards, does not always bathe or change clothes even if prompted. Poor grooming has caused social ostracism at school/residence/work, or required intervention. Eating erratic and poor, may require medical intervention.

7 Extremely Severe
Most areas of hygiene/appearance/nutrition are extremely poor and easily noticed as below usual community standards OR hygiene/appearance/nutrition requires urgent and immediate medical intervention.

14. DISORIENTATION: Does not comprehend situations or communications, such as questions asked during the entire BPRS interview. Confusion regarding person, place, or time. Do not rate if incorrect responses are due to delusions.

May I ask you some standard questions we ask everybody?
How old are you? What is the date (allow + or - 2 days)? What is this place called? What year were you born? Who is the president?

1 Not Present

2 Very Mild
Seems muddled or mildly confused 1 - 2 times during interview. Oriented to person, place, and time.

3 Mild
Occasionally muddled or mildly confused 3 - 4 times during interview. Minor inaccuracies in person, place, or time, e.g., date off by more than + or - 2 days, or gives wrong division of hospital.

4 Moderate
Frequently confused during interview. Minor inaccuracies in person, place, or time are noted, as in "3" above. In addition, may have difficulty remembering general information, e.g., name of president.

5 Moderately Severe
Markedly confused during interview, or to person, place, or time. Significant inaccuracies are noted, e.g., date off by more than one week, or cannot give correct name of hospital. Has difficulty remembering personal information, e.g., where he/she was born, or recognizing familiar people.

6 Severe
Disoriented to person, place, or time, e.g., cannot give correct month and year. Disoriented in 2 out of 3 spheres.

7 Extremely Severe
Grossly disoriented to person, place, or time, e.g., cannot give name or age. Disoriented in all 3 spheres.

Rate items 15 - 24 on the basis of observed behavior and speech.

15. CONCEPTUAL DISORGANIZATION: Degree to which speech is confused, disconnected, vague or disorganized. Rate tangentiality, circumstantiality, sudden topic shifts, incoherence, derailment, blocking, neologisms, and other speech disorders. Do not rate content of speech.

1 Not Present

2 Very Mild
Peculiar use of words or rambling but speech is comprehensible.

3 Mild
Speech a bit hard to understand or make sense of due to tangentiality, circumstantiality or sudden topic shifts.

4 Moderate
Speech difficult to understand due to tangentiality, circumstantiality, idiosyncratic speech, or topic shifts on many occasions OR 1-2 instances of incoherent phrases.

5 Moderately Severe
Speech difficult to understand due to circumstantiality, tangentiality, neologisms, blocking, or topic shifts most of the time OR 3-5 instances of incoherent phrases.

6 Severe
Speech is incomprehensible due to severe impairments most of the time. Many BPRS items cannot be rated by self report alone.

7 Extremely Severe
Speech is incomprehensible throughout interview.

16. BLUNTED AFFECT: Restricted range in emotional expressiveness of face, voice, and gestures. Marked indifference or flatness even when discussing distressing topics. In the case of euphoric or dysphoric patients, rate Blunted Affect if a flat quality is also clearly present.

Use the following probes at end of interview to assess emotional responsivity:

Have you heard any good jokes lately? Would you like to hear a joke?

1 Not Present

2 Very Mild
Emotional range is slightly subdued or reserved but displays appropriate facial expressions and tone of voice that are within normal limits.

3 Mild
Emotional range overall is diminished, subdued, or reserved, without many spontaneous and appropriate emotional responses. Voice tone is slightly monotonous.

4 Moderate
Emotional range is noticeably diminished, patient doesn't show emotion, smile, or react to distressing topics except infrequently. Voice tone is monotonous or there is noticeable decrease in spontaneous movements. Displays of emotion or gestures are usually followed by a return to flattened affect.

5 Moderately Severe
Emotional range very diminished, patient doesn't show emotion, smile or react to distressing topics except minimally, few gestures, facial expression does not change very often. Voice tone is monotonous much of the time.

6 Severe
Very little emotional range or expression. Mechanical in speech and gestures most of the time. Unchanging facial expression. Voice tone is monotonous most of the time.

7 Extremely Severe
Virtually no emotional range or expressiveness, stiff movements. Voice tone is monotonous all of the time.

17. EMOTIONAL WITHDRAWAL: Deficiency in patient's ability to relate emotionally during interview situation. Use your own feeling as to the presence of an "invisible barrier" between patient and interviewer. Include withdrawal apparently due to psychotic processes.

1 Not Present

2 Very Mild

Lack of emotional involvement shown by occasional failure to make reciprocal comments, occasionally appearing preoccupied, or smiling in a stilted manner, but spontaneously engages the interviewer most of the time.

3 Mild
Lack of emotional involvement shown by noticeable failure to make reciprocal comments, appearing preoccupied, or lacking in warmth, but responds to interviewer when approached.

4 Moderate
Emotional contact not present much of the interview because subject does not elaborate responses, fails to make eye contact, doesn't seem to care if interviewer is listening, or may be preoccupied with psychotic material.

5 Moderately Severe
Same as "4" but emotional contact not present most of the interview.

6 Severe
Actively avoids emotional participation. Frequently unresponsive or responds with yes/no answers (not solely due to persecutory delusions). Responds with only minimal affect.

7 Extremely Severe
Consistently avoids emotional participation. Unresponsive or responds with yes/no answers (not solely due to persecutory delusions). May leave during interview or just not respond at all.

18. MOTOR RETARDATION: Reduction in energy level evidenced by slowed movements and speech, reduced body tone, decreased number of spontaneous body movements. Rate on the basis of observed behavior of the patient only. Do not rate on the basis of patient's subjective impression of his own energy level. Rate regardless of the medication effects.

1 Not Present

2 Very Mild
Slightly slowed or reduced movements or speech compared to most people.

3 Mild
Noticeably slowed or reduced movements or speech compared to most people.

4 Moderate
Large reduction or slowness in movements or speech.

5 Moderately Severe
Seldom moves or speaks spontaneously OR very mechanical or stiff movements.

6 Severe
Does not move or speak unless prodded or urged.

7 Extremely Severe
Frozen, catatonic.

19. TENSION: Observable physical and motor manifestations of tension, "nervousness," and agitation. Self-reported experiences of tension should be rated under the item on anxiety. Do not rate if restlessness is solely akathisia, but do rate if akathisia is exacerbated by tension.

1 Not Present

2 Very Mild
More fidgety than most but within normal range. A few transient signs of tension, e.g., picking at fingernails, foot wagging, scratching scalp several times, or finger tapping.

3 Mild
Same as "2," but with more frequent or exaggerated signs of tension.

4 Moderate

Many and frequent signs of motor tension with one or more signs some times occurring simultaneously, e.g., wagging one's foot while wringing hands together. There are times when no signs of tension are present.

5 Moderately Severe
Many and frequent signs of motor tension with one or more signs often occurring simultaneously. There are still rare times when no signs of tension are present.

6 Severe
Same as "5," but signs of tension are continuous.

7 Extremely Severe
Multiple motor manifestations of tension are continuously present, e.g., continuous pacing and hand wringing.

20. UNCOOPERATIVENESS: Resistance and lack of willingness to cooperate with the interview. The uncooperativeness might result from suspiciousness. Rate only uncooperativeness in relation to the interview, not behaviors involving peers and relatives.

1 Not Present

2 Very Mild
Shows nonverbal signs of reluctance, but does not complain or argue.

3 Mild
Gripes or tries to avoid complying, but goes ahead without argument.

4 Moderate
Verbally resists but eventually complies after questions are rephrased or repeated.

5 Moderately Severe
Same as 4, but some information necessary for accurate ratings is withheld.

6 Severe

Refuses to cooperate with interview, but remains in interview situation.

7 Extremely Severe
Same as 6, with active efforts to escape the interview.

21. EXCITEMENT: Heightened emotional tone, or increased emotional reactivity to interviewer or topics being discussed, as evidenced by increased intensity of facial expressions, voice tone, expressive gestures or increase in speech quantity and speed.

1 Not Present

2 Very Mild
Subtle and fleeting or questionable increase in emotional intensity. For example, at times seems keyed up or overly alert.

3 Mild
Subtle but persistent increase in emotional intensity. For example, lively use of gestures and variation in voice tone.

4 Moderate
Definite but occasional increase in emotional intensity. For example, reacts to interviewer or topics that are discussed with noticeable emotional intensity. Some pressured speech.

5 Moderately Severe
Definite and persistent increase in emotional intensity. For example reacts to many stimuli, whether relevant or not, with considerable emotional intensity. Frequent pressured speech.

6 Severe
Marked increase in emotional intensity. For example reacts to most stimuli with inappropriate emotional intensity. Has difficulty settling down or staying on task. Often restless, impulsive, or speech is often pressured.

7 Extremely Severe
Marked and persistent increase in emotional intensity. Reacts to all stimuli with inappropriate intensity, impulsiveness. Cannot settle down or stay on task. Very restless and impulsive most of the time. Constant pressured speech.

22. DISTRACTIBILITY: Degree to which observed sequences of speech and actions are interrupted by stimuli unrelated to the interview. Distractibility is rated when the patient shows a change in the focus of attention or a marked shift in gaze. Patient's attention may be drawn to noise in adjoining room, books on shelf, interviewer's clothing, etc. Do not rate circumstantiality, tangentiality, or flight of ideas. Also, do not rate rumination with delusional material. Rate even if the distracting stimulus cannot be identified.

1 Not Present

2 Very Mild
Generally can focus on interviewer's questions with only 1 distraction or inappropriate shift of attention of brief duration.

3 Mild
Patient shifts focus of attention to matters unrelated to the interview 2-3 times.

4 Moderate
Often responsive to irrelevant stimuli in the room, e.g., averts gaze from the interviewer.

5 Moderately Severe
Same as above, but now distractibility clearly interferes with the flow of the interview.

6 Severe
Extremely difficult to conduct interview or pursue a topic due to preoccupation with irrelevant stimuli.

7 Extremely Severe

Impossible to conduct interview due to preoccupation with irrelevant stimuli.

23. MOTOR HYPERACTIVITY: Increase in energy level evidenced in more frequent movement and/or rapid speech. Do not rate if restlessness is due to akathisia.

1 Not Present

2 Very Mild
Some restlessness, difficulty sitting still, lively facial expressions, or somewhat talkative.

3 Mild
Occasionally very restless, definite increase in motor activity, lively gestures, 1-3 brief instances of pressured speech.

4 Moderate
Very restless, fidgety, excessive facial expressions or nonproductive and repetitious motor movements. Much pressured speech, up to one third of the interview.

5 Moderately Severe
Frequently restless, fidgety. Many instances of excessive non-productive and repetitious motor movements. On the move most of the time. Frequent pressured speech, difficult to interrupt. Rises on 1-2 occasions to pace.

6 Severe
Excessive motor activity, restlessness, fidgety, loud tapping, noisy, etc. throughout most of the interview. Speech can only be interrupted with much effort. Rises on 3-4 occasions to pace.

7 Extremely Severe
Constant excessive motor activity throughout entire interview, e.g., constant pacing, constant pressured speech with no pauses, interviewee can only be interrupted briefly and only small amounts of relevant information can be obtained.

24. MANNERISMS AND POSTURING: Unusual and bizarre behavior, stylized movements or acts, or any postures which are clearly uncomfortable or inappropriate. Exclude obvious manifestations of medication side effects. Do not include nervous mannerisms that are not odd or unusual.

1 Not Present

2 Very Mild
Eccentric or odd mannerisms or activity that ordinary persons would have difficulty explaining, e.g., grimacing, picking. Observed once for a brief period.

3 Mild
Same as "2," but occurring on two occasions of brief duration.

4 Moderate
Mannerisms or posturing, e g., stylized movements or acts, rocking, nodding, rubbing, or grimacing, observed on several occasions for brief periods or infrequently but very odd. For example, uncomfortable posture maintained for 5 seconds more than twice.

5 Moderately Severe
Same as "4," but occurring often, or several examples of very odd mannerisms or posturing that are idiosyncratic to the patient.

6 Severe
Frequent stereotyped behavior, assumes and maintains uncomfortable or inappropriate postures, intense rocking, smearing, strange rituals, or fetal posturing. Subject can interact with people and the environment for brief periods despite these behaviors.

7 Extremely Severe
Same as "6," but subject cannot interact with people or the environment due to these behaviors.
